# A Correction

**Published:** 1893-01

**Authors:** G. Jaeger


### A CORRECTION.

[From *Allg. Hom. Zeitung*, Vol. 125, p. 157.]

I have been told lately that many find my theory of potentiation too "materialistic." This is remarkable. A medicine is

*matter*, and *dead* matter at that. How is it possible to present a theory of the action of matter otherwise than as "materialistic"? Nobody can doubt that a *physical* phenomenon must be explained *physically*, an *optical* one optically, an *acoustic* one acoustically, hence, also, a *materialistic* phenomenon must be explained *materialistically*. I am the last to contend that in a *living being* also other *non-materialistic* processes take place, still less do I doubt that, in treating a patient, non-materialistic potencies can play a great rôle; yea, that a patient can be cured entirely with such. But one thing is incontestable: nobody ever thought of potentiating the spirit by shaking it with alcohol, but this has always been done only with *material substances*, and hence an explanation of the changes which the matter undergoes thereby cannot by any means be otherwise than materialistic, for if it is not, then it is nothing.

PROF. DR. G. JAEGER.

